# A case of multiple perforating pilomatricomas^☆^

**DOI:** 10.1016/j.abd.2020.09.015

**Published:** 2022-02-10

**Authors:** Mai Endo, Toshiyuki Yamamoto

**Affiliations:** Department of Dermatology, Fukushima Medical University, Fukushima, Japan

Dear Editor,

A 22-year-old man was referred to our department for evaluation of a slightly painful nodule on the arm that had increased in size over the previous four months. He had a history of surgical resection of several nodules on the nape, back, and arm at other dermatology clinics. He complained of myotonic symptoms with difficulty in relaxing his hands after contraction and was followed at the Neurology Department of our hospital under suspicion of myotonic dystrophy, but he refused to undergo genetic testing. Physical examination showed a 10 × 10 mm, slightly elevated, firm reddish nodule with central brownish crusts on the left upper arm (Fig. 1a). The tumor was surgically removed under local anesthesia. Histopathological examination showed a well-circumscribed tumor mass penetrating the dermis. The epidermis was ulcerated, and the lateral acanthotic epidermis extended downwards in a tongue-like projection (Fig. 1b). Tumor cells were composed of different types of cells, such as eosinophilic, basophilic, and transitional cells. Partial elimination of tumor cells through the epidermis was observed (Fig. 1b). There was an inflammatory reaction with mononuclear cells, and a number of foreign-body type giant cells were found around the mass. We retrospectively examined three specimens of previously resected tumors. One of the specimens also showed proliferation of eosinophilic and basaloid tumor cells in the dermis with central ulceration of the epidermis (Fig. 1c), whereas the other two tumors were pilomatricoma without perforation.

**Figure 1:**
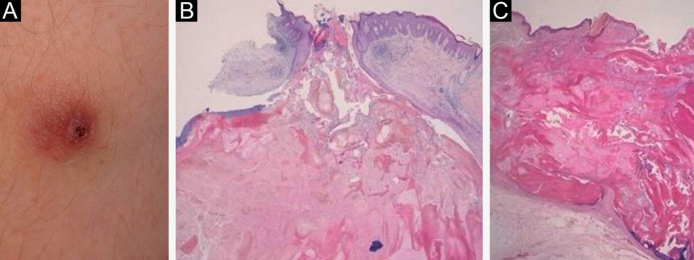
(A), A reddish firm nodule with central crusts on the upper arm. (B), A perforated pilomatricoma lesion with elimination of pilomatricoma cells (Hematoxylin & eosin stain; ×40). (C), Histopathology of another nodule, showing central ulceration and an eosinophilic tumor mass with peripheral basaloid tumor cells (Hematoxylin & eosin stain; ×40).

Perforating pilomatricoma is a rare variant of pilomatricoma, which clinically presents with inflammatory papules, cutaneous horn-like nodules, craters, and ulcers resembling keratoacanthoma or solid masses.^1^, ^2^, ^3^, ^4^ Most of the reported cases are solitary lesions, and one case has been reported in which perforating pilomatricoma was observed in one of the multiple pilomatricomas;^2^ however, to our knowledge, only one case of multiple onset of perforating pilomatricomas has been reported.^3^ In that case, the patient presented Churg-Strauss syndrome and Rubinstein-Taybi syndrome and developed a total of 15 tumors. Although the exact number was not specified, multiple lesions were classified as perforating pilomatricomas.^3^ Our patient was suspected of myotonic dystrophy, which, however, was not genetically confirmed. Myotonic dystrophy is an autosomal dominant inherited disease caused by a mutation in the dystrophia myotonica protein kinase gene on chromosome 19. The occurrence of multiple pilomatricomas has been reported to be associated with myotonic dystrophy.^5^ Our patient developed multiple firm nodules on the nape, back, and upper arm. The total number of the nodules was five, four of which were subjected to histopathological examination. All of the four nodules were diagnosed as pilomatricomas. Of interest, two of the nodules were perforating pilomatricomas, showing perforation and partial elimination of the eosinophilic tumor cells. Recent studies have shown that matrix metalloproteinase-9 and -12 derived from tumor cells, fibroblasts, and macrophages may be relevant to the partial elimination of the tumor, by degradation of collagen and elastic fibers.^4^ When superficially located, the elimination of pilomatricoma is accelerated by virtue of epidermal catharsis mechanisms.

## Financial support

None declared.

## Authors’ contributions

Mai Endo: Designed the study; performed the research and contributed to analysis and interpretation of data; wrote the initial draft of the manuscript; read and approved the final version of the manuscript.

Toshiyuki Yamamoto: Designed the study; performed the research and contributed to analysis and interpretation of data; assisted in the preparation of the manuscript; read and approved the final version of the manuscript.

## Conflicts of interest

None declared.
